# Supplementary material for: Mycobacterium tuberculosis infection in pregnancy: A systematic review
Source: PLOS Glob Public Health. 2024 Nov 22;4(11):e0003578. doi: 10.1371/journal.pgph.0003578 (PMC11584094; doi:10.1371/journal.pgph.0003578)
Supplement: S2 Appendix — (DOCX) [file pgph.0003578.s002.docx]

**S2 Appendix. Newcastle-Ottawa Scale (Modified Version)**

**Conflicts of Interest**

No evidence of conflicts of interest by one or more authors

1. Yes
2. No

**Selection**

Representative of the exposed cohort

1. Yes

0. No

Cohort characteristics are well described.

1. Yes

0. No

Ascertainment of the exposure is well described.

1. Yes

0. No

**Comparability**

If heterogeneous groups were included in the study cohort, results were disaggregated by these characteristics.

1. Yes

0. No

**Outcome**

Ascertainment of outcome is well described.

1. Yes

0. No

Was follow-up long enough for outcomes to occur?

1. Yes

0. No

Adequacy of follow-up of cohort - follow-up of ≥80% of participants

1. Yes

0. No

**Overall study quality score**

0-2 Poor

3-5 Fair

6-8 Good
